# Proteomic profiling of serum in cats with naturally occurring degenerative joint disease and co-morbid conditions

**DOI:** 10.3389/fpain.2025.1501932

**Published:** 2025-02-04

**Authors:** B. Duncan X. Lascelles, Rakesh Ponnala, Steven G. Kamerling, Tracey Williams

**Affiliations:** ^1^ Translational Research in Pain, Department of Clinical Sciences, College of Veterinary Medicine, North Carolina State University, Raleigh, NC, United States; ^2^ Comparative Pain Research and Education Center, North Carolina State University, Raleigh, NC, United States; ^3^ Thurston Arthritis Center, UNC School of Medicine, Chapel Hill, NC, United States; ^4^ Center for Translational Pain Research, Department of Anesthesiology, Duke University, Durham, NC, United States; ^5^ Veterinary Medicine Research & Development, Zoetis, Kalamazoo, MI, United States; ^6^ Veterinary Pharmacology Consultant, Veterinary Medicine Research & Development, Zoetis, Kalamazoo, MI, United States

**Keywords:** feline, degenerative joint disease (DJD), osteoarthritis (OA), chronic kidney disease (CKD), pain

## Abstract

**Introduction:**

Degenerative joint disease (DJD) occurs very commonly in cats and can be associated with pain. Almost 70% of cats with DJD-associated pain suffer the co-morbidity of chronic kidney disease (CKD). There are currently very limited treatment or management options. A greater understanding of the systems biology of DJD, DJD-associated pain, and CKD may contribute to identifying disease specific biomarkers and relevant targets for the development of therapeutics for the control of these conditions in cats, and help inform human pain therapeutic development.

**Methods:**

Using mass spectrometry-based proteomic profiling of the serum of 200 highly phenotyped cats with varying burdens of DJD, pain, and CKD, we identified significant individual proteins and pathways.

**Results:**

Functional pathway analysis, based on differentially abundant proteins across individual disease states (DJD, pain, CKD), identified pathways playing a role in DJD and DJD-associated pain including acute phase response signaling, LXR/RXR and FXR/RXR activation and the complement system. With the added co-morbidity of CKD, similar pathways were identified, with the addition of IL-12 signaling and production in macrophages.

**Discussion:**

We identified differentially abundant proteins associated with DJD, pain and CKD and future work should evaluate these proteins as potential biomarkers of disease (individually or as clusters). Further, these data could be leveraged to identify novel therapeutic targets to address the gap in our ability to manage DJD, pain, and CKD in cats. Given that our work was in cats with naturally occurring DJD, these results may have translational applicability to human health.

## Introduction

Little is understood about the global processes involved in driving degenerative joint disease (DJD), the pain associated with DJD, or chronic kidney disease (CKD) in cats. Previous studies by Marino et al. (1) showed a high co-prevalence of CKD and DJD, and suggested there may be common mechanisms across these conditions. In the present study, we aimed to advance our understanding of mechanisms involved in these individual disease states, and potentially identify disease specific biomarkers, by using proteomic profiling of serum from highly phenotyped cats spanning the spectra of disease severity. This is the first study performed in cats, and the first study in any species that includes full phenotyping with every joint radiographed and every joint assessed for pain status. Serum proteomic analysis of human patients with and without osteoarthritis (OA) has been performed, but generally in relation to OA of specific joints (2–4). No work has been performed using a cohort of human patients that have been fully characterized in terms of total joint disease burden and pain burden.

We used feline serum samples collected from cats with DJD and/or CKD. Detailed clinical data were available for each cat, including age, body condition score, disease stage/category, and pain category.

We used Hyper Reaction Monitoring (HRM) mass spectrometry (MS) based on a SWATH-MS type data independent acquisition (DIA) approach for the proteomic profiling of these samples [developed by, and performed by Biognosys AG (5)]. This approach allowed for the quantification of all detectable peptide signals in one single shot HRM measurement per sample. Spectral libraries (“peptide inventories”) were generated using high-performance data dependent acquisition (DDA) on pooled samples according to sample groups for targeted extraction of the DIA data (6).

Raw protein intensity data from samples was filtered, scaled, and normalized. Evaluation of differential protein expression was conducted using statistical methods to identify dysregulated proteins across specific disease state and severity. Further, pathway analysis was conducted on the dysregulated proteins which identified significant pathways for each specific disease state.

We hypothesized that through identifying individual proteins or groups of proteins associated with DJD, DJD-associated Pain, or CKD (or combinations of these factors) we would in turn identify underlying disease mechanisms, potential therapeutic targets (proteins or pathways), and potential disease specific biomarkers. Furthermore, we hypothesized that inflammation or immune dysfunction may be a common mechanism underlying both DJD and CKD and the pain associated with these diseases.

## Methods

### Sample source

Samples used in this study were banked serum samples that had been collected from cats presented to the Translational Research in Pain (TRiP) Program at North Carolina State College of Veterinary Medicine (formally called Comparative Pain Research Laboratory) over the period from May 2007 to May 2015. The metadata used were data collected at the time of the original study. All data were collected at the time of “screening” for potential study inclusion, and all data were cross-sectional and from a single point in time. The work described in this report was performed in 2017. Each original (source) study and procedures were individually approved by the North Carolina State University College of Veterinary Medicine Institutional Animal Care and Use Committee (IACUC) (ethical approval committee). All methods in the original studies (7–10) were performed in accordance with the IACUC approval. All original studies were reported using ARRIVE or CONSORT guidelines. The cats included in this study represented a mix of cats that presented for several studies, including those that were actively recruited as normal controls (where there was a requirement for no owner-noted mobility impairment) and those that presented for studies of treatments for DJD (7–10). Across all these studies, the cats had been evaluated in the same manner. Cats recruited to these DJD studies were required to have owner-noted mobility impairment based on validated owner questionnaires (7, 11). All cats had been examined by a veterinarian, and had been evaluated for systemic disease, orthopedic pain, and radiographic evidence of DJD, as previously described (7, 8, 12, 13). Briefly, all cats received a physical examination, including evaluation of body condition score (BCS) (14), followed by an orthopedic examination during which each joint or spinal segment was palpated and gently manipulated, and scored for the presence and severity of pain. Pain was scored on the following scale: 0 = no resentment; 1 = mild withdrawal, mild resistance to manipulation; 2 = moderate withdrawal, body tenses, may orient to site, may vocalize or increase vocalization; 3 = orients to site, forcible withdrawal from manipulation, may vocalize, hiss, or bite; and 4 = tries to escape or prevent manipulation, bites or hisses, marked guarding of site (13). Total pain (TPain) scores were calculated as the sum of the scores for individual joints, with a possible range of 0–80 [as previously described (13)] and were further categorized for some analyses as described in the Statistical Analysis section.

Following the physical and orthopedic examinations, cats were sedated and digital radiographs were made of each joint and spinal segment. Radiographs were evaluated by a single investigator (BDXL) who was unaware of the results of the physical and orthopedic examination, and radiographs were scored for the presence and severity of DJD using previously published criteria established by Lascelles' laboratory—the Translational Research in Pain Program (8). Scores were ascribed according to a 10-point scale where 0 = no evidence of DJD and 10 = ankylosis of the joint. Total radiographic DJD (TDJD) scores were calculated as the sum of the scores for individual joints, with a possible range of 0–200 (as previously described (8)) and were further categorized for some analyses as described in the Statistical Analysis section.

For both DJD scores and pain scores, “severity categories” of normal, low, moderate and high were created based on the following, as described previously (15): TPain scores were categorized as 0–2 = negligible/normal (as long as no single joint received a score of 2); 2–4 = low (a score of 2 was placed in this category if a single joint received a score of 2); 5–9 = moderate; ≥10 = high. TDJD scores were categorized as 0–3 = negligible/normal; 4–12 = low; 13–24 = moderate; ≥25 = high.

Prior to sedation, all cats had urine samples collected by cystocentesis for urinalysis, and blood samples collected for a complete blood count, serum biochemistry analysis, and for sample archiving. Sample collection typically occurred following the physical examination and prior to the orthopedic examination. For serum samples, whole blood was collected into a sterile 3 ml anti-coagulant free plastic tube and allowed to clot at room temperature for at least 30 min, but no more than 60 min. Clotted samples were centrifuged at 1,163×*g* for 10 min, and serum was removed, aliquoted, and stored in cryovials at −80 °C until use. Based on results from the serum biochemistry, urinalysis, and review of radiographs and previous medical records, cats were classified as CKD positive (+ve) or negative (−ve) according to the guidelines set forth by the International Renal Interest Society (16) and described in Marino et al. (1). Briefly, and as described in Appendix 1 of Marino et al. (1) complete blood count, serum chemistry, urinalysis, and radiographic determination of degenerative kidney changes and kidney size, were used to designate cats as CKD+ve or CKD−ve. If the features of parenchymal mineralization, irregular margins and/or misshapen kidneys were seen radiographically, then cats were considered to have degenerative kidney changes. Kidney size was determined as described in Marino et al. (1). Urine specific gravity (USG) is not a reliable indicator of feline kidney function and so cats with a USG 1.013–1.034 and without other indicators of kidney disease were categorized as CKD−ve. Cats with CKD were staged according to the International Renal Interest Society (IRIS) guidelines; however, without urine protein to creatinine ratios and blood pressure measurements, IRIS substaging was not possible in this study. Sample collections were performed when cats were not on treatment—prior to any treatments being administered. In addition, all cats were required to be either naïve to treatment for pain or have had a 4-week washout period prior to presentation.

“Disease states” were considered “DJD”, “Pain” and “CKD”. DJD and Pain were further broken into categories (normal, low, moderate, high—see above), and CKD was dichotomized into present or absent (CKD+ve; CKD−ve).

Stored serum samples were eligible for inclusion in this study if they had been collected from cats that had received a physical and orthopedic examination, and radiographic evaluation of each joint and spinal segment. Further inclusion criteria specified that the samples had been maintained at −80 °C and had not been through any freeze-thaw cycles prior to testing.

### Overview of protein identification methods

Two hundred serum samples from included cats were prepared for mass spectrometry (protein extraction and tryptic digestion to peptides) using Biognosys' (Biognosys, Schlieren, Switzerland) optimized sample preparation protocol.

#### Sample preparation

All solvents were high performance liquid chromatography (HPLC)-grade from Sigma-Aldrich and all chemicals, unless stated otherwise, were obtained from Sigma-Aldrich. Biognosys' denature buffer was added to 200 samples of 10 µl cat serum. Samples were reduced and alkylated using Biognosys' reduction and alkylation buffers. Subsequently, samples were digested overnight with sequencing grade modified trypsin (Promega). C18 cleanup for MS was carried out according to the manufacturer's instructions using C18 96 well plates (Nest Group Inc., Southborough, MA, USA). Peptide solutions were evaporated to complete dryness using a SpeedVac system. Dried peptides were dissolved in Biognosys' liquid chromatography (LC) solvent and Biognosys' HRM calibration kit peptides were added. The final peptide concentrations were determined for all samples by measurement of absorbance at 280 nm (Spectrostar^NANO^, BMG Labtech). To create a Spectral Library (see section below), randomly selected MS ready samples (peptides) from low + normal and moderate + high DJD categories were pooled for fractionation (two pools). High pH reversed-phase peptide fractionation was performed on C18 MicroSpin columns (Nest Group Inc., Southborough, MA, USA) and 6 fractions were obtained per pool (elution with 5%, 10%, 15%, 20%, 25% and 50% acetonitrile at pH 10). Fractions 5% and 50% were combined for the measurement. QC pool samples were generated by combining equal aliquots of all individual peptide preparations.

#### Generation of mass spectrometry data and data analysis

A spectral library was generated by performing liquid chromatography—tandem mass spectrometry (LC-MS/MS) (shotgun) measurements of the fractionated pools of samples (low + normal pool and moderate + high pool). Injections of 2 µg quantities of peptides per fraction were made into an in-house packed C18 column (Dr. Maisch ReproSil Pur, 1.9 µm particle size, 120 Å pore size; 75 µm inner diameter, 50 cm length, New Objective) on a Thermo Scientific Easy nLC 1200 nano-LC system connected to a Thermo Scientific Q Exactive MS equipped with a standard nano-electrospray source. LC solvents were: (A) 1% acetonitrile in water with 0.1% formic acid, and (B) 15% water in acetonitrile with 0.1% formic acid. The non-linear LC gradient was 1%–55% solvent B in 120 min followed by 55%–90% B in 2 min, and 90% B for 12 min (total gradient length of 134 min). A modified TOP12 method from Olsen was used (17). LC-MS/MS datasets were analyzed using the MaxQuant 1.5.6.5 software package and searches were performed against the Felis_catus_6.2 Ensembl database, allowing for two missed cleavages and including N-terminal acetylation and methionine oxidation modifications, asparagine/glutamine deamidation, lysine/arginine carbamylation. False discovery rate (FDR) for peptide and protein levels was set to one percent.

For the proteomic profiling using Biognosys' HRM technology (based on a DIA approach), 5 µg of peptides per individual sample and QC pools were injected to an C18 column (CSH-C18 1,7 µm, 300 µm inner diameter, 150 mm length) on a Waters M-Class LC connected to a Thermo Scientific Fusion Lumos MS equipped with a next gen nanoFlex electrospray source. LC solvents were: (A) 1% acetonitrile in water with 0.1% formic acid, and (B) 15% water in acetonitrile with 0.1% formic acid. The nonlinear LC gradient was 1%–49% solvent B in 40 min followed by steps of 90% B for 1 min, and 1% B for 4 min. A DIA method with one full range survey scan and 29 DIA windows was used. Integrity of MS raw files was checked by lab personnel using viewer software before accepting the run. Data processing was performed in Spectronaut software (version 10, Biognosys), and each peak was scored based on a range of parameters (i.e., peak shape, mass accuracy, isotope distribution, coelution with MS1, etc.). A combined score (CScore) was then evaluated using a target/decoy model (Scrambled CMID decoy settings), and a q-value filter of 0.01 was applied on precursor and protein level. Missing values were imputed using empirical imputation in Spectronaut (Sparse setting). A complete data matrix was generated, containing all identified proteins with relative quantities across samples. Median% CVs for replicate injections of QC samples over the acquisition sequence was 13.2% on precursor level and 17.3% on protein level.

### Process of normalization of data (scaling proteomics data from biognosys; filtering, data normalization, and differential protein expression)

Data manipulation and plotting was done using R statistical software. Protein to gene mapping was performed based on annotations from an ENSEMBL protein database for cat (Felis_catus_6.2.pep.all.fasta). Mapping to corresponding human gene and protein identifications was performed using BLAST (Protein-Protein BLAST 2.2.27 + against Homo sapiens proteome Uniprot/SPROT version 2013-04-29). Data were first normalized to reduce inter-experimental variance, using local regression normalization (18) as implemented in Spectronaut software. Values were then scaled to dimensions compatible with transcriptomics analysis. Proteins with scaled intensity <5 were filtered out and the remaining proteins were normalized by using the DESeq2 Bioconductor Software Package and a geometric mean method across all samples. To detect differentially abundant proteins (DAPs), we used the DESeq (19) method, by fitting samples to a negative binomial generalized linear model for each protein followed by a Wald statistical test for significance between individual disease states and severity categories. To correct for multiple testing, *p*-values were adjusted using the Benjamini-Yekutieli procedure to control the FDR (20). Proteins with a *p*-value <0.05 were considered significant in each category and further prioritized for pathway analysis.

### Pathway analysis

Pathway analysis was performed for proteins (DAPs) for categories of DJD + Pain, DJD + Pain and DJD + Pain + CKD using Ingenuity Pathway Analysis (IPA; https://www.qiagenbioinformatics.com/products/ingenuity-pathway-analysis) software. We performed core analysis on DAPs from these categories. Specifically, we identified canonical pathways, upstream regulators, toxicological functions, and protein networks. Top canonical pathways from each of the categories where prioritized by statistical significance cutoff of −log (*p*-value) > 3.0 for DJD + Pain, and −log (*p*-value) > 2.0 for DJD + Pain and DJD + Pain + CKD.

## Results

The demographics of the cats are detailed in Table 1, and the number of cats in each severity category of each disease state are detailed in Table 2.

**Table 1:** Demographics of the cats from which samples were obtained and analyzed.

Variable	Mean (±SD)	Range
Age (years)	11.32 (±3.8)	1.4–19.3
Weight (kg)	5.52 (±1.42)	2.63–11.52
BCS	6.5 (±1.6)	1–9
Total DJD score	18.6 (±13.5)	0–73
Total Pain score	13.6 (±6.7)	0–34
Sex distribution		
Sex	FS = 112	
	MC = 88	
Breed distribution (number)		
Abyssinian	1	
British shorthair	1	
DLH	25	
DMH	4	
DSH	137	
Himalayan	2	
Maine coon	8	
Manx	3	
Oriental shorthair	1	
Persian	3	
Ragdoll	2	
Rex	2	
Scottish fold	1	
Siamese	10	

BCS, body condition score; DJD, degenerative joint disease; FS, female spayed; MC, male castrated; DLH, domestic long hair; DMH, domestic medium hair; DSH, domestic short hair; SD, standard deviation.

**Table 2:** Severity category for each disease state (assessed in 200 cats) based on score.

Disease state	Score ranges	Severity category	Number of cats
DJD	0–3	Normal	27
	4–12	Low	50
	13–24	Moderate	59
	≥25	High	64
Pain	0–1	Normal	11
	2–4	Low	10
	6–9	Moderate	23
	≥10	High	156
CKD*	n/a	Negative	133
	n/a	Positive	67

This includes individual cats with single or multiple disease states. The raw data on disease state and severity category for each cat are provided in Supplementary Table 1. Of the 200 cats, 23 had no radiographic evidence of DJD at all and were negative for CKD; however, 11 of these had moderate to high total pain scores. This illustrates that, not unexpectedly, the cats in this cohort exhibited spectrums of condition severity.

* Of the 67 CKD positive cats, 9 were IRIS stage 1; 57 were IRIS stage 2; and 1 was IRIS stage 3.

As CKD progresses the filtration efficiency of the kidneys declines impacting the abundance of circulating proteins in the blood. For example, smaller proteins (less than 30 kDa) which would normally be filtered out of the body via the kidneys, may accumulate in circulation due to reduced clearance of waste products and small regulatory proteins [e.g., creatinine (approximately 113 Da); β2-microglobulin (approximately 12 kDa)] or may be filtered excessively if the glomerular filtration barrier is damaged [e.g., retinol binding protein 4 (21 kDa)]; circulating levels of intermediate proteins (30–100 kDa) may be altered due to disrupted tubular reabsorption and metabolic changes [e.g., α-1-microglobulin (approximately 33 kDa); erythropoietin (approximately 30.4 kDa)], and in the presence of a compromised glomerular filtration barrier, large proteins (more than 100 KDa) which would normally be retained in circulation [immunoglobulins (about 150 kDa); fibronectin (approximately 440 kDa)] can leak into the urine, leading to proteinuria.

Therefore, some of the proteins presented in this proteomic analysis may not be directly associated with DJD and the Pain associated with DJD but could be an outcome of kidney function compromise. Further work should evaluate a larger cohort of cats with no comorbidities to add confidence to the findings.

### Differentially abundant proteins across DJD and pain disease states and severity categories

**Figure 1:**
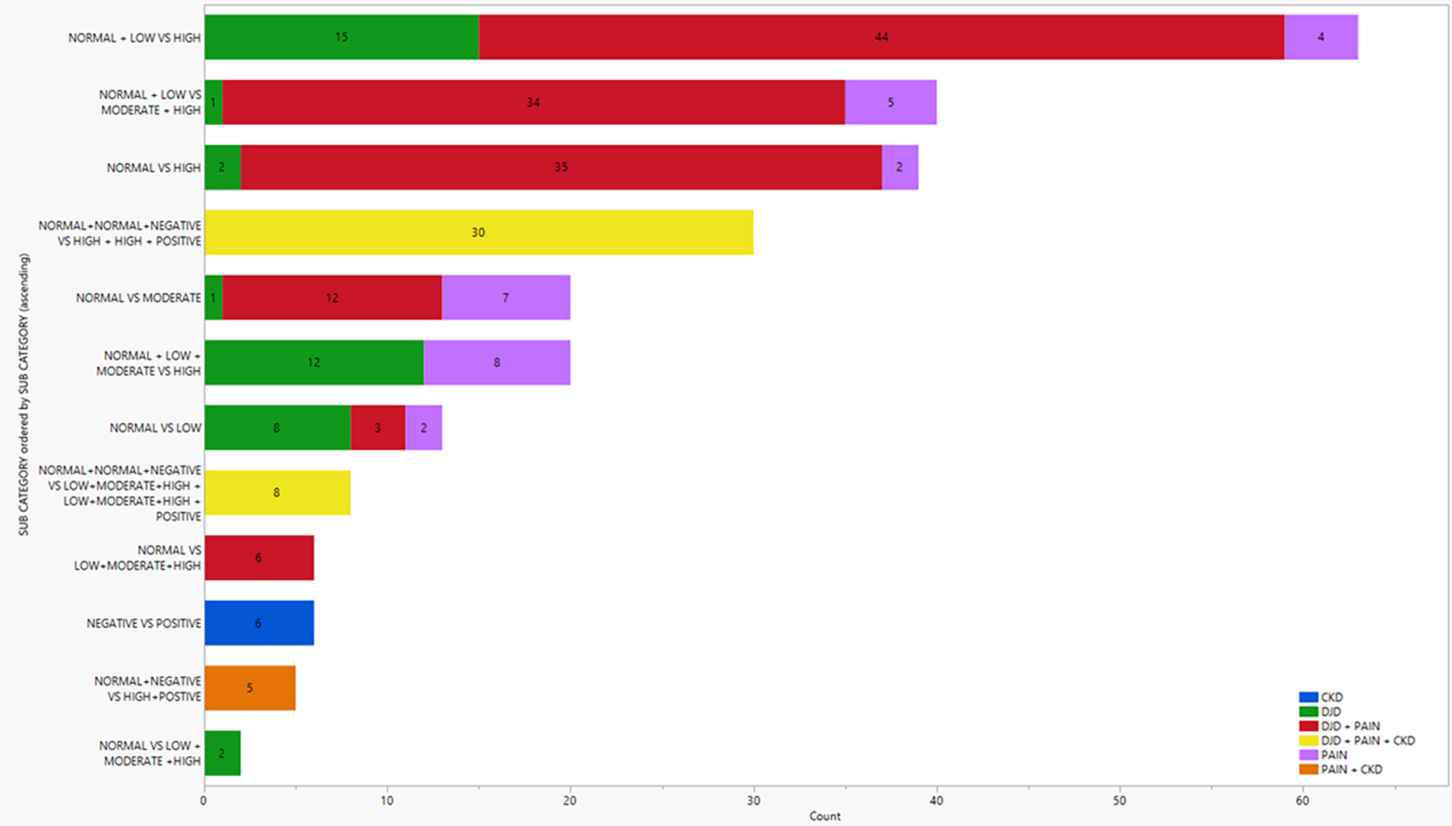
Illustration of the number of differentially abundant proteins in various disease states and severity category combinations. Colors represent the different disease states. Normal, low, moderate and high relate to the severity of DJD and Pain; negative and positive relate to the absence or presence of CKD.

Differential protein abundance analysis was carried out on all disease states and categories of severity as shown in Figure 1 (and see Supplementary Table 2 for individual fold changes). As part of our analysis, severity categories were grouped and compared among disease states. Similarly, disease states were grouped and compared among severities. These results are depicted in Figure 1. We found a higher number of differentially abundant proteins (DAPs) in three severity category grouping comparisons (normal + low vs. high; normal + low vs. moderate + high; normal vs. high). In each of these severity category groupings, the referenced cats had higher severities of DJD and/or pain. The highest number of DAPs were seen in the high severity categories of “DJD + Pain”, shown in red on the top three horizontal bars in Figure 1.

**Figure 2:**
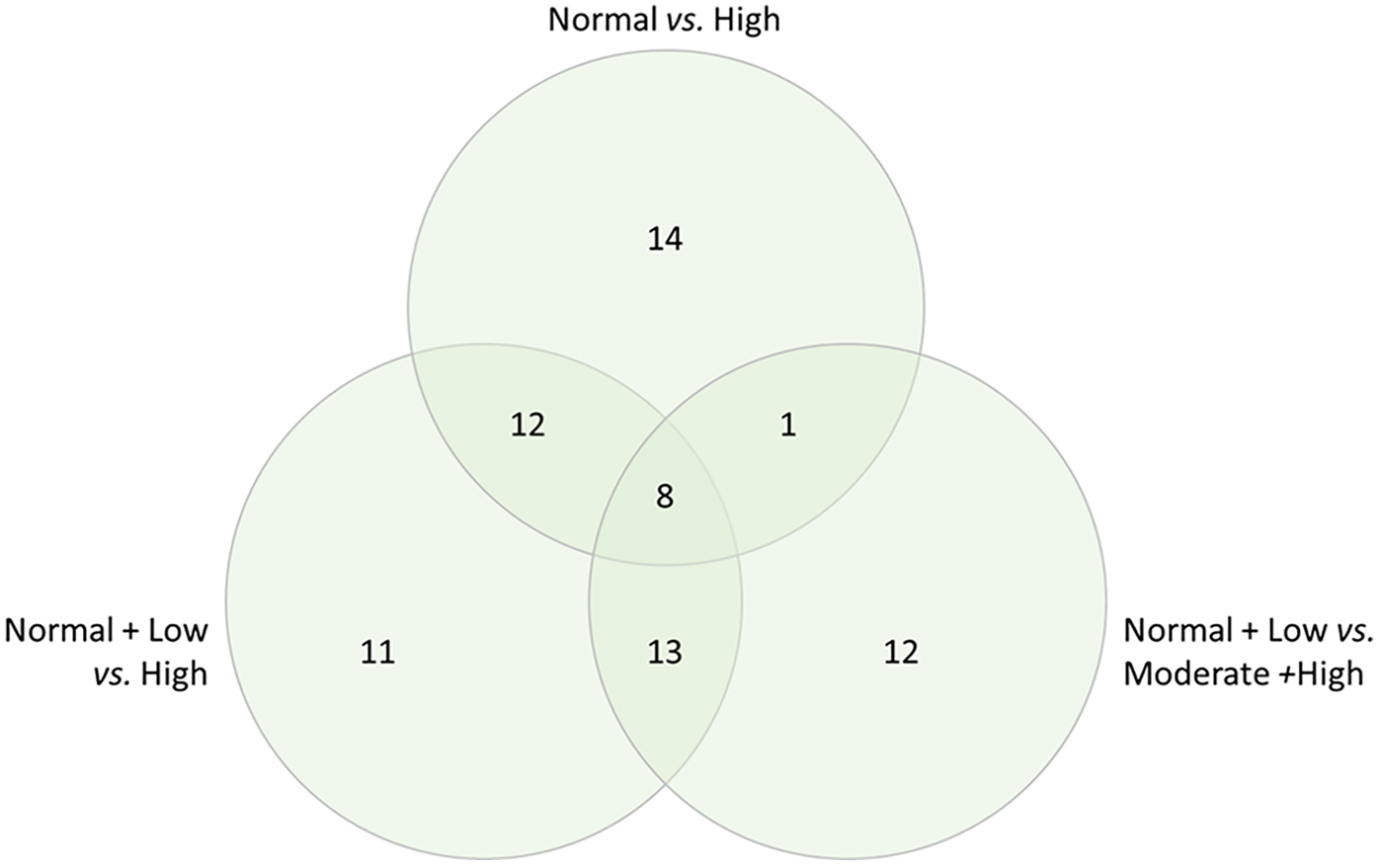
Differentially abundant proteins in each severity category combination, and overlap, for the combined disease states of DJD + pain, illustrated in a venn diagram.

This suggested that DAPs due to the disease states of DJD + Pain might have an effect in both the disease states and might play a crucial role in the biology. Taking the DAPs for each DJD + Pain severity category combination where we saw the highest number of DAPs (normal + low vs. high; normal + low vs. moderate + high; normal vs. high), we identified the DAPs in each severity category combination, and the overlap between severity category combinations. The number of DAPs in each severity category combination and the overlap is shown in Figure 2 (and see Supplementary Table 2 for individual proteins for each category).

The DAPs (*n* = 8) common to the three major groupings of disease severity are indicated in Table 3.

**Table 3:** Differentially abundant proteins common to (seen in all) the three major comparisons of severity category (normal + low vs. high; normal + low vs. moderate + high; normal vs. high) for DJD and Pain disease states.

Protein	Protein description	Protein molecular weight (KDa)	Location	Dysregulation
AP3D1	Adaptor related protein complex 3 subunit delta 1	166.5	Cytoplasm	Downregulated
ASMTL	Acetylserotonin O-methyltransferase like	35.1	Cytoplasm	Upregulated
C1QB	Complement component subcomponent subunit B	28.3	Extracellular Space	Upregulated
FETUB	Fetuin B	38.6	Extracellular Space	Upregulated
GOSR2	Golgi SNAP receptor complex member 2	26.1	Cytoplasm	Upregulated
KRT77	Keratin 77	51.1	Cytoplasm	Downregulated
C3orf17	Nucleolus and neural progenitor protein	64.6	Nucleus	Upregulated
PLTP	Phospholipid transfer protein	61.9	Extracellular Space	Upregulated

### Differentially abundant proteins across all disease states (DJD, pain, CKD) and severity categories

**Figure 3:**
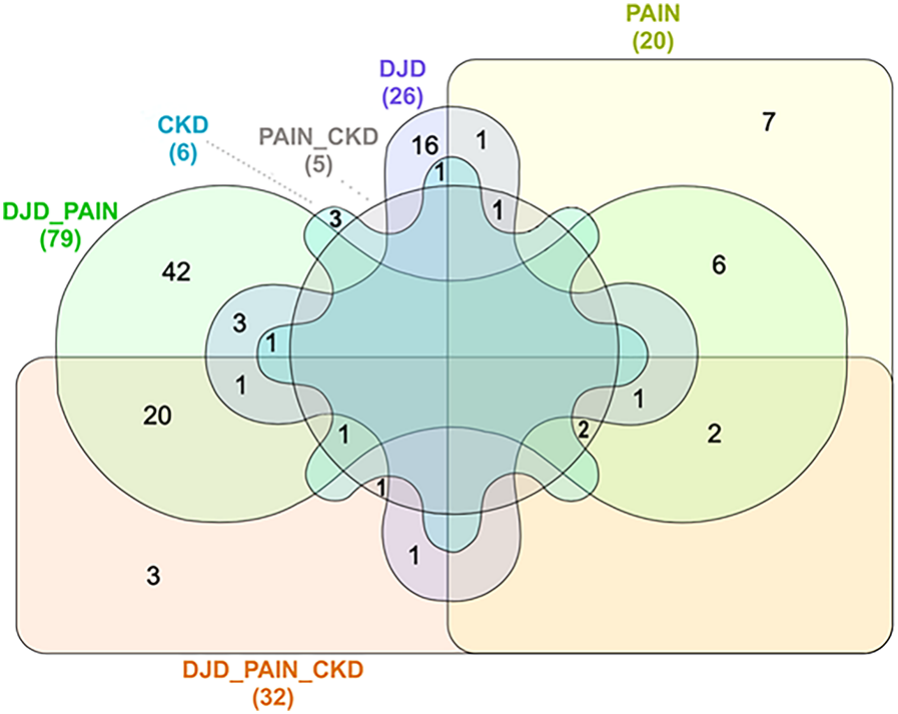
Venn diagram illustration of numbers of distinct differentially abundant proteins by disease category, with overlap illustrated.

Next, using all severity categories within a disease state, the number of DAPs for each disease state, and overlapping disease states, were defined. These results are illustrated in Figure 3 (and see Supplementary Table 2 for individual proteins for each category). As expected, the highest number of DAPs (i.e., 79) was seen in the combination of DJD + Pain disease states, although the highest fold changes in DAPs were seen in the Pain disease state (data not shown). The second highest number of overlapping DAPs was observed for DJD + Pain + CKD (i.e., 32), followed by Pain + CKD (i.e., 5). The individual proteins identified are tabulated in Table 4 for each disease state.

**Table 4:** Table detailing the differentially abundant proteins in each disease state.

Disease state	Number of proteins	Dysregulation	
		Upregulated	Downregulated
DJD	16	ANTXR1 (59.9), DUSP2 (17.9), ANKAR (33.7), CNOT3 (138.1), PSMA5 (30.1), HBB (15.9), HBE1 (16.3), HBA1 (15.6), HERC6 (142.2), CFHR3 (42.9), VTN (67.3)	ABTB2 (69.2), PPP1R21 (57.1), PSMB1 (28.3), MAK (83.3), GTF3C2 (112.3)
PAIN	7	CRTAC1 (28.5), ENSFCAP00000022519 (13.9*), KRT1-201 (66.2), DPYSL5 (31.9), EMR4P (50.9^†^), KRT71 (48.9)	SAA1 (11.8)
CKD	3	TBC1D4 (140.2), CTSB (37.6), SERPINF1 (46.3)	

ABTB2, ankyrin repeat and BTB/POZ domain-containing protein 2; ANKAR, ankyrin and armadillo repeat-containing protein; ANTXR1, Anthrax toxin receptor 1; CFHR3, complement factor H-related protein 3; CNOT3, CCR4-NOT transcription complex subunit 3; CRTAC1, cartilage Acidic Protein 1; CTSB, cathepsin B; DPYSL5, dihydropyrimidinase Like 5; DUSP2, dual specificity protein phosphatase 2; EMR4P, putative EGF-like module-containing mucin-like hormone receptor-like 4; ENSFCAP00000022519, protein not annotated; GTF3C2, general transcription factor 3C polypeptide 2; HBA1, hemoglobin subunit alpha; HBB, hemoglobin subunit beta; HBE1, hemoglobin subunit epsilon; HERC6, probable E3 ubiquitin-protein ligase HERC6; KRT1-201, keratin 1; KRT71, keratin; type II cytoskeletal 71; MAK, serine/threonine-protein kinase MAK; PPP1R21, protein phosphatase 1 regulatory subunit 21; PSMA5, proteasome subunit alpha type-5; PSMB1, proteasome subunit beta type-1; SAA1, serum amyloid A-1 protein; TBC1D4, TBC1 domain family member 4; SERPINF1, pigment epithelium-derived factor; VTN, vitronectin. Protein molecular weight (KDa) is shown in brackets after each protein.

* Estimate based on amino acid sequence.

^†^ Predicted.

### Protein network for DJD and pain

**Figure 4:**
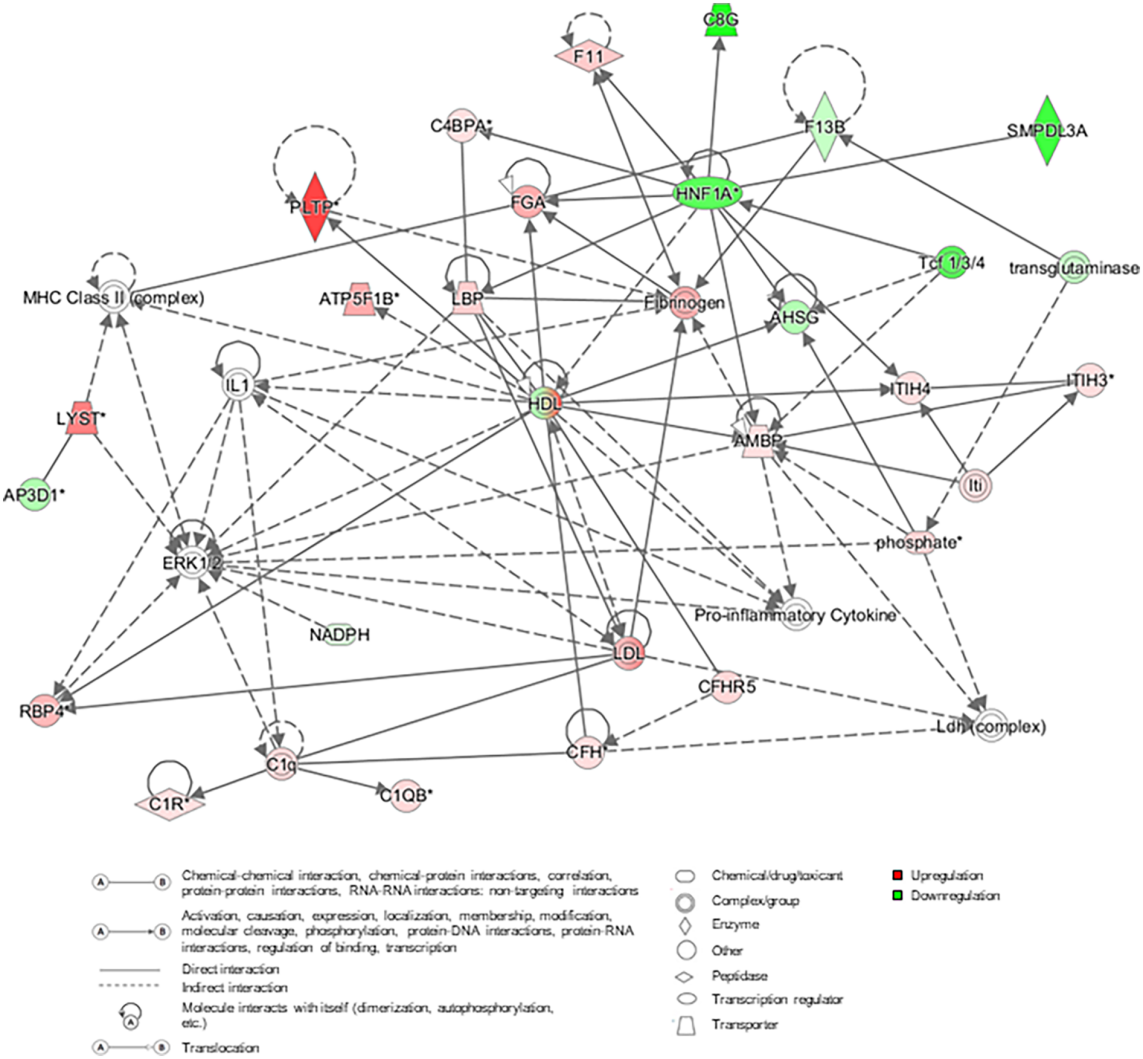
Protein network constructed using the differentially abundant proteins from the three severity category groupings for DJD + pain (normal + low vs. high; normal + low vs. moderate + high; normal vs. high). In the network, red and pink indicates that proteins are upregulated, green that proteins are downregulated, and grey colored proteins are those that have been added from literature (human, mouse and rat) based on interactions. AHSG, alpha-2-HS-glycoprotein; AMBP, protein AMBP; AP3D1, AP-3 complex subunit delta-1; ATP5B, ATP synthase F1 subunit beta; C1q, complement C1q; C1QB, complement C1q subcomponent subunit B; C1R, complement C1r subcomponent; C4BPA, C4b-binding protein alpha chain; C8G, complement component C8 gamma chain; CFH, complement factor H; CFHR5, complement factor H-related protein 5; ERK, extracellular signal-regulated kinases; F11, coagulation factor XI; F13B, coagulation factor XIII B chain; FGA, fibrinogen alpha chain; HDL, high-density lipoprotein; HNF1A, hepatocyte nuclear factor 1-alpha; IL1, interleukin 1; Iti, inter-alpha-trypsin inhibitor; ITIH3, inter-alpha-trypsin inhibitor heavy chain H3; ITIH4, inter-alpha-trypsin inhibitor heavy chain H4; LBP, lipopolysaccharide-binding protein; LDL, low-density lipoprotein; LYST, lysosomal-trafficking regulator; MHC, major histocompatibility complex; NADPH, nicotinamide adenine dinucleotide phosphate; PLTP, phospholipid transfer protein; RBP4, retinol-binding protein 4; SMPDL3A, sphingomyelin phosphodiesterase acid Like 3A; Tcf, T cell factor.

A protein network was built from DAPs across the DJD + Pain severity categories (normal + low vs. high; normal + low vs. moderate + high; normal vs. high) using IPA, and this is illustrated in Figure 4. This network illustrates the interconnectivity of proinflammatory components identified in the DJD and Pain disease states.

### Pathway analysis for DJD and pain proteins

To further understand the systems biology of the proteins, we carried out functional and pathway analysis on DAPs from the three severity category groupings with the highest number of DAPs (illustrated in Figure 1).

Using the proteins identified as being differentially abundant in each of the three disease severity category comparisons for DJD + Pain (normal + low vs. high; normal + low vs. moderate + high; normal vs. high), pathway analysis was carried out. The top pathways identified were: Acute Phase Response Signaling, Liver X Receptor (LXR)/Retinoid X Receptor (RXR) Activation, Farnesoid X Receptor (FXR)/RXR Activation, and Complement System.

**Figure 5:**
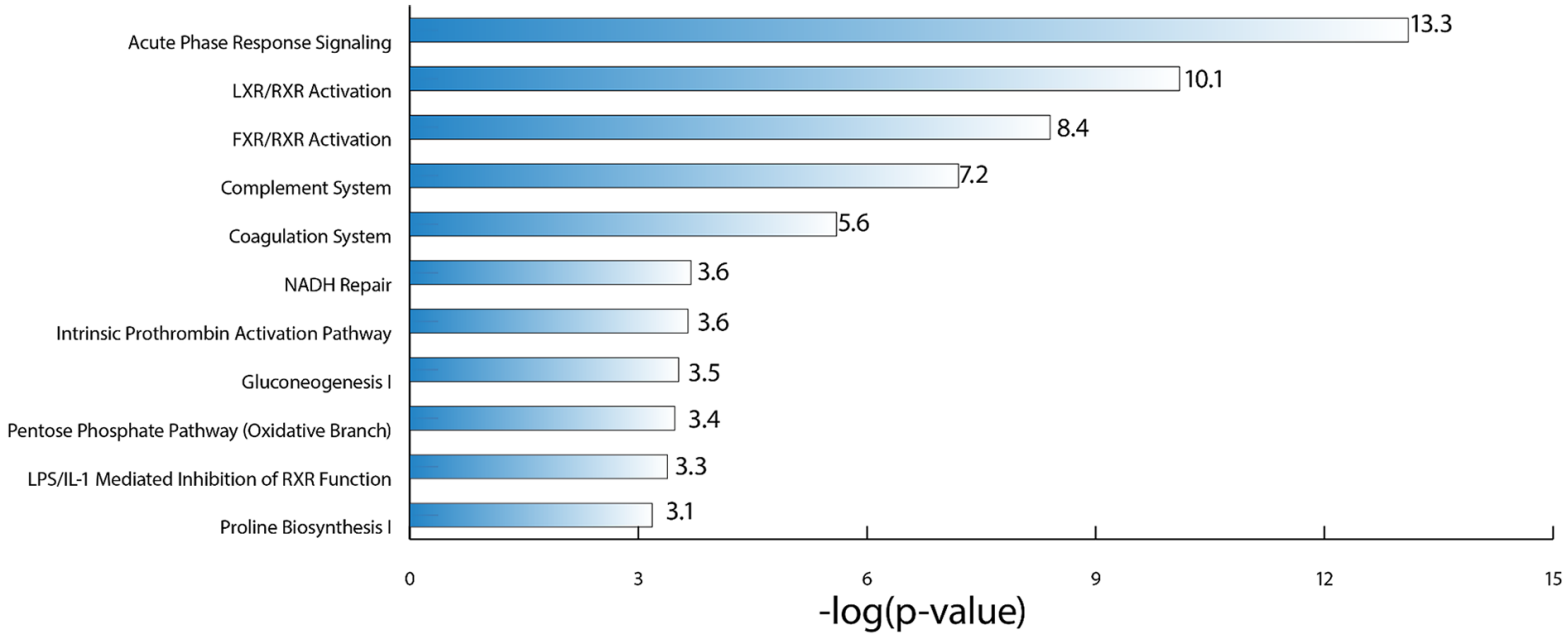
Illustration of the top canonical pathways, based on log *P*-value, that were identified as being perturbed in the DJD and pain disease states, using the differentially abundant proteins from the three severity category groupings for DJD and pain (normal + low vs. high; normal + low vs. moderate + high; normal vs. high).

Together, these pathways suggest a role of macrophages, the complement system, and lipid metabolism in the liver in DJD + Pain. The top five pathways identified based on log-*P* value are illustrated in Figure 5, and the associated DAPs are tabulated in Table 5.

**Table 5:** Alphabetical list of the differentially abundant proteins (DAPs) from the three disease severity category comparisons for DJD + pain (normal + low vs. high; normal + low vs. moderate + high; normal vs. high) that were identified as driving perturbation of individual canonical pathways.

Canonical pathways	−log (*p*-value)	Dysregulation	
		Upregulated	Downregulated
Acute phase response signaling	13.3	AMBP (34.8), C1R (80.9), C4BPA (187.4), FGA (53.9), ITIH3 (26.6), ITIH4 (25.9), LBP (57.8), RBP4 (21.5), VWF (39.6)	AHSG (46.7), HNF1A (63.3), IL1RAP (57.2)
LXR/RXR activation	10.1	AMBP (34.8), FGA (53.9), ITIH4 (25.9), LBP (57.8), PLTP (61.9), RBP4 (21.5), S100A8 (10.8)	AHSG (46.7), IL1RAP (57.2)
FXR/RXR activation	8.4	AMBP (34.8), FETUB (38.6), FGA (53.9), ITIH4 (25.9), PLTP (61.9), RBP4 (21.5)	AHSG (46.7), HNF1A (63.3)
Complement system	7.2	C1QB (28.3), C1R (80.9), C4BPA (187.4), CFH (151.2)	C8G (73.7)
Coagulation system	5.6	F11 (58.9), FGA (53.9), VWF (39.6)	F13B (71.3)

AHSG, alpha-2-HS-glycoprotein; AMBP, protein AMBP; C1QB, complement C1q subcomponent subunit B; C1R, complement C1r subcomponent; C4BPA, C4b-binding protein alpha chain; C8G, complement component C8 gamma chain; CFH, complement factor H; F11, coagulation factor XI; F13B, coagulation factor XIII B chain; FETUB, fetuin-B; FGA, fibrinogen alpha chain; HNF1A, hepatocyte nuclear factor 1-alpha; IL1RAP, interleukin-1 receptor accessory protein; ITIH3, inter-alpha-trypsin inhibitor heavy chain H3; ITIH4, inter-alpha-trypsin inhibitor heavy chain H4; LBP, lipopolysaccharide-binding protein; PLTP, phospholipid transfer protein; RBP4, retinol-binding protein 4; S100A8, S100 calcium binding protein A8; VWF, von Willebrand factor. Protein molecular weight (KDa) is shown in brackets after each protein.

### Common pathways between DJD + pain and DJD + pain + CKD

**Figure 6:**
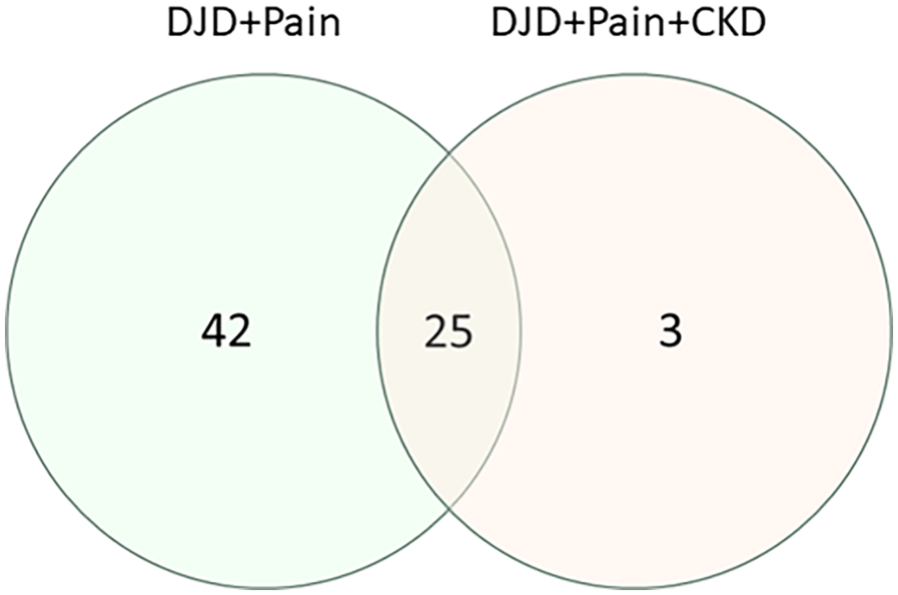
Venn diagram illustrating how the numbers of differentially abundant proteins from the three disease severity groupings for DJD + pain (normal + low vs. high; normal + low vs. moderate + high; normal vs. high) overlap with the differentially abundant proteins from a group of disease severity for DJD + Pain + CKD (normal + normal + negative vs. high + high + positive).

Differentially abundant proteins in the three groupings of DJD + Pain disease severity were compared with the DAPs from one group of DJD + Pain + CKD disease severity (normal + normal + CKD-ve vs. high + high + CKD + ve). A very high proportion of DAPs in the DJD + Pain + CKD disease states were found to overlap with DJD + Pain (Figure 6 and see Supplementary Table 2 for individual proteins for each category). The top pathways identified were FXR/RXR Activation, LXR/RXR Activation, Acute Phase Response Signaling, IL12 Signaling and Production in Macrophages, and LPS/IL-1 Mediated Inhibition of RXR Function (Table 6).

**Table 6:** Commonly perturbed (upregulated or downregulated) pathways in the DJD + pain and DJD + pain + CKD disease states.

Canonical pathways	−log (*p*-value)	Direction of dysregulation	
		Upregulation	Downregulation
LXR/RXR activation	5.05	PLTP (61.9), RBP4 (21.5)	AHSG (46.7), IL1RAP (57.2)
FXR/RXR activation	4.98	FETUB (38.6), PLTP (61.9), RBP4 (21.5)	AHSG (46.7)
Acute phase response signaling	3.01	RBP4 (21.5)	AHSG (46.7), IL1RAP (57.2)
LPS/IL-1 mediated inhibition of RXR function	2.73	FABP4 (15.4), PLTP (61.9)	IL1RAP (57.2)
IL-12 signaling and production in macrophages	2.04	MST1R (150.4), RBP4 (21.5)	

AHSG, alpha-2-HS-glycoprotein; FABP4, fatty acid-binding protein; adipocyte; FETUB, fetuin-B; IL1RAP, interleukin-1 receptor accessory protein; MST1R, macrophage-stimulating protein receptor; PLTP, phospholipid transfer protein; RBP4, retinol-binding protein 4. Protein molecular weight (KDa) is shown in brackets after each protein.

**Figure 7:**
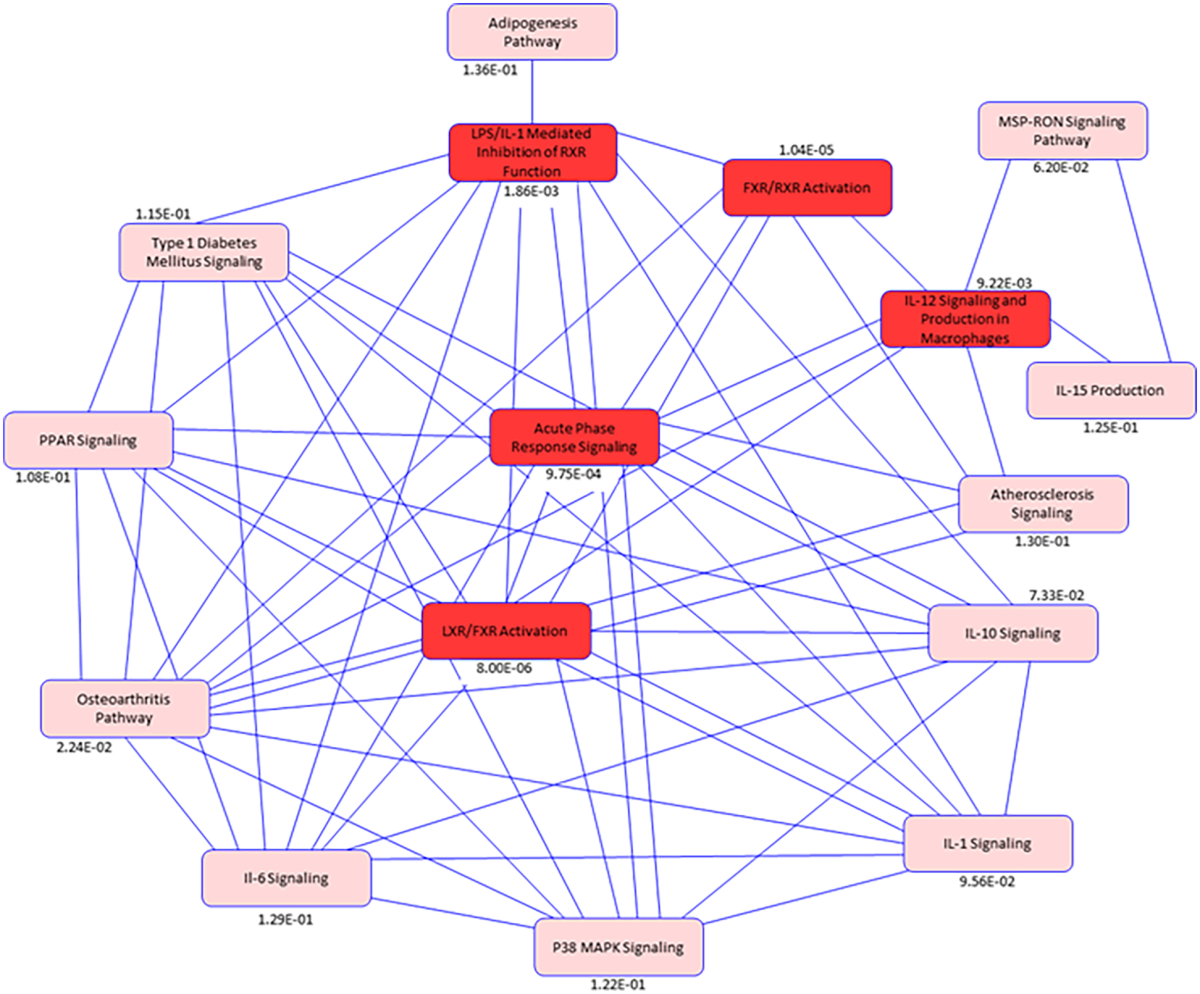
Pathway network diagram. Illustration of pathway analysis results for the differentially abundant proteins common to the disease states of DJD, Pain and CKD. Blue Color intensity refers to the degree of connectivity of a pathway with other pathways between the disease conditions (DJD, Pain, CKD). Highlighted Red colors are common pathways identified in the disease states. While pink colors indicates pathways identified from publicly available datasets in humans, mice, and rats that are biologically linked to the pathways identified in this feline data, The *p*-value for each pathway is listed below individual pathway boxes.

To identify the connectivity between pathways and the underlying common mechanisms between disease states, we performed network analysis among the top pathways. Our pathway network indicates (Figure 7) among the top pathways, Acute Phase Response Signaling was the most interconnected pathway with other common pathways, suggesting the critical role of macrophages and immune-mediated responses in both DJD + Pain and DJD + Pain + CKD disease states.

## Discussion

### Individual proteins DJD + pain

The highest number of DAPs were seen in the DJD + Pain disease state. Of the 8 DAPs common to the three major groupings of disease severity (Table 3), four [adaptor related protein complex 3 subunit delta 1 (AP3D1); golgi SNAP receptor complex member 2 (GORS2); keratin 77 (KRT77); and nucleolus and neural progenitor protein (C3orf17)] have not been described as being associated with pain or osteoarthritis (OA) in the literature. Acetylserotonin O-methyltransferase like protein (ASMTL) is involved in the production of melatonin (21) and was upregulated (log_2_ fold change = 0.21–0.22) in the DJD + Pain state. Melatonin has analgesic properties (21) but there are no reports discussing ASMTL in pain conditions or OA. If ASMTL plays a role in DJD and pain via melatonin, its upregulation may be a compensatory mechanism. Complement component 1q subunit B (C1Qb) was upregulated (log_2_ fold change = 0.14) in the DJD + Pain categories in our study. C1Qb, and indeed the complement pathway in general, has been implicated in neuropathic pain. Expression of the gene encoding for C1Qb was increased in spinal cord tissue after peripheral nerve ligation (22). This has been confirmed in several subsequent reports (23), and DJD-associated pain is believed to be partly due to neuropathic pain processes. Upregulation of the complement system is known to play a role in the disease of OA (24), and specifically, C1Qb has also been implicated. Complement component 1q (C1q) protein was shown to be abundant and secreted by human articular chondrocytes and to bind to chondrocytes to negatively influence relative collagen expression (25). Fetuin B (FETUB) is an inhibitor of proteases and has been found to be increased in the serum of both humans with OA (3) and in dogs following cruciate ligament transection (26). In our study, it was upregulated (log_2_ fold change = 0.39–0.47) in the DJD + Pain disease state. As a serum glycoprotein, the physiological role of FETUB is poorly understood, but it shares some functions with fetuin-A (FETUA). FETUA plays a role in many physiological aspects, such as fatty acid transport, response to systemic inflammation, osteogenesis and bone resorption, and inhibition of calcification. FETUB, similarly to FETUA, is an inhibitor of basic calcium phosphate precipitation, but its potential role in OA has not been investigated. There are no reports of the role of FETUB in pain. Plasma phospholipid transfer protein (PLTP) mediates both net transfer and exchange of phospholipids between different lipoproteins. We found PLTP to be upregulated (log_2_ fold change = 0.46–0.69) in the DJD + Pain disease state, consistent with recent literature describing a role of PLTP in joint inflammation. Recently, a role in inflammation, independent of its role in phospholipid transfer, has been described. PLTP was found to be highly abundant in the joints of rheumatoid arthritis (RA) patients and it was suggested it may directly trigger inflammation and fibroblast-like synoviocyte (FLS) proliferation, independent of its lipid transfer activity (27). In that study, PLTP was not as highly abundant in OA synovium from patients, but there were no control tissues to compare expression in OA synovium. FLS from OA patients responded in a similar way to those from RA patients, with increases in production of inflammatory cytokines (27).

### Differentially abundant proteins in each disease state and disease state

For the individual disease states, several proteins were differentially abundant (Table 4). For some, there is a known role in the disease state, and for others, no role has been reported. The DAPs for each disease state are discussed below where it is considered they may play a potential role.

#### DJD disease state

##### Upregulated proteins in the DJD disease state

Anthrax toxin receptor 1 (ANTXR1; log_2_ fold change = 0.59–0.65) is highly abundant in cartilaginous tissue and is upregulated by runt-related transcription factor 2 (Runx2) (28). ANTXR1 regulates chondrocyte proliferation and overexpression of ANTXR1 causes chondrocyte apoptosis and matrix mineralization. Apoptosis is an important factor in OA and Runx2 is a causative molecule in OA (28). Members of the dual specificity phosphatase (DUSP) subfamily can directly dephosphorylate the members of the mitogen-activated protein kinase superfamily [extracellular signal-regulated kinases (ERK), c-Jun N-terminal kinases, and p38], and several members of the DUSP subfamily have anti-inflammatory functions. DUSP5 has been shown to suppress interleukin (IL)-1 beta (1β) induced chondrocyte inflammation (29). We found DUSP2 was upregulated in DJD (log_2_ fold change = 0.57–0.61) and as such, this may be a compensatory change. Vitronectin (VTN; log_2_ fold change = 0.15) promotes cell adhesion and inhibits terminal complement components. VTN was upregulated in synovial fluid from pigs that underwent untreated anterior cruciate ligament transection (30). Serum concentrations of VTN fragments were elevated in serum from OA patients and subsequent work corroborated this and indicated a pro-OA role for VTN fragments via integrin (31). CCR4-NOT complex (CNOT3; log_2_ fold change = 0.49–0.53) inhibits bone resorption by downregulating receptor activator of nuclear factor kappa-*Β*, but there have been no reports linking dysregulation of this protein to OA. Proteasome subunit alpha type-5 (PSMA5; log_2_ fold change = 0.44–0.74) controls ubiquination and proteolysis of cellular proteins. No association with DJD or OA has been reported.

##### Down regulated proteins in the DJD disease state

No role in, or association with, DJD or OA has been reported for any of the downregulated proteins except for complement factor H-related 3 (CFHR3; log_2_ fold change = −0.37). The role of CFHR3 in inflammation and OA are discussed below in Complement Pathway section.

#### Pain disease state

##### Upregulated proteins in the pain disease state

Cartilage acidic protein 1 (CRTAC1; log_2_ fold change = 0.68) was recently identified and detected in the synovial fluid of patients with OA by proteomic analysis (32). Further work by the same group showed that CRTAC1 is induced by pro-inflammatory cytokines in articular chondrocytes and synovial fibroblasts and deletion of *crtac1* gene in female mice inhibited the development of OA (histologically), and restored gait parameters indicative of pain, to normal (33). Recently, plasma proteomic studies have found plasma CRTAC1 was correlated with both disease severity (34) and joint pain (35, 36) in human patients with OA, and was found to predict progression to joint replacement (35, 36). Its role is poorly understood, and a direct link to neurobiological processes driving pain has not been described. No roles for keratin 71 (KRT71; log_2_ fold change = 1.03) or keratin 1 (KRT1-201; log_2_ fold change = 0.65) in pain have been described in the literature. Dihydropyrimidinase-related protein 5 (DPYSL5; log_2_ fold change = 1.14) is thought to play a role in neural development, but no links to pain are described, other than antibodies to this protein, which have been described in association with chemotherapy-induced neuropathic pain. Adhesion G protein-coupled receptor E4 (EMR4P; log_2_ fold change = 1.09) is abundant on activated macrophages, and is involved in leukocyte adhesion and migration (37, 38). Overexpression of the gene encoding for EMR4P was recently described as a novel finding in a study looking at whole blood gene expression signatures across the co-existing conditions of asthma, dermatitis, and rhinitis (39).

##### Down regulated proteins in the pain disease state

Interestingly, serum amyloid A1 (SAA1) was down-regulated (log_2_ fold change = −0.69), yet studies have shown that SAA1 induces expression of pro-inflammatory cytokines (such as IL-1β, tumor necrosis factor alpha (TNFα), and matrix metalloproteinases (MMP-1 and MMP-13) (40).

### CKD disease state

Serpin family F member 1 (SERPINF1) is a member of the serine protease inhibitor family with antiangiogenic, antioxidative, anti-inflammatory, antitumorigenic, and renoprotective activity (41) It was upregulated (log_2_ fold change = 0.12) in cats with CKD correlating with human data in which SERPINF1 has been shown to increase in later stages of CKD (42). Cathepsin B (CTSB) is a lysosomal cysteine protease involved in the degradation of lysosomal proteins and was upregulated (log_2_ fold change = 0.49) in cats with CKD. With cellular damage, released CTSB has been proposed to contribute to multiple pathological conditions including inflammatory disease, arthritis, and nephropathy and has been shown to be associated with age related reduction in kidney function in women (43). Our data showed that Tre-2, BUB2, CDC16, 1 domain family member 4 (TBC1D4;AS160), a Rab-GTPase activating protein, was upregulated (log_2_ fold change = 0.54) in cats with CKD. TBC1D4 is involved in glucose homeostasis and has been shown to control glucose transporter type 4 (GLUT4) in adipose and skeletal muscle cells (44), and is known to be associated with diabetic nephropathy (45).

### Protein networks

A protein network was built from DAPs in the DJD and Pain categories using IPA.

Several significant hubs appear in the Protein Network involving lipid metabolism, coagulation, the complement cascade, and cytokine production. The liver is likely orchestrating these interactions. Lipid metabolism/transport and liver involvement are supported by the numerous connections between high-density lipoprotein and low-density lipoprotein proteins and inflammatory cytokines (e.g., IL-1, lipopolysaccharide binding protein) complement components, fibrinogen, alpha-1-microglobulin (AMBP), hepatocyte nuclear factor 1 homeobox A and PLTP. Specific interconnections between IL-1, coagulation, complement, and microglobulins retinol binding protein (RBP)/AMBP support the involvement of these proteins/pathways in DJD and pain as well as CKD. The liver has perhaps been underappreciated as a regulatory organ in the pathogenesis of inflammation and progression of DJD and CKD.

There are smaller but potentially significant contributions from (1) T-cell transcription factor associated with Wnt activation in OA (46), (2) sphingomyelin phosphodiesterase associated with lipid metabolism and immune cell regulation (47) and (3) FETUA associated with biomineralization of bone (48). The preponderance of inputs to ERK is consistent with the role of this kinase in the promotion of inflammatory events at the cellular level. It is worth noting that not all disease-promoting genes were upregulated. Some were downregulated, which is likely a compensatory response and an attempt to restore homeostasis.

### Pathways

Our work identified several pathways that were perturbed in the DJD + Pain disease state, or both in the DJD + Pain and DJD + Pain + CKD disease states. These are discussed below.

#### Acute phase response (APR) signaling

The APR is stimulated by the release of cytokines such as IL-1, IL-6, and TNFα from macrophages and monocytes at the site of inflammatory lesions or infections. This results in changes in serum acute phase proteins (APP), primarily synthesized by hepatocytes, but also by extra-hepatic tissues. The APR may result in changes in more than 200 proteins grouped as either positive APP or negative APP (49). The proteins we identified as up- and down-regulated in our study of chronic DJD and pain in cats generally agreed with the published information on the APR and APP, in particular, the increase in complement and coagulation proteins. Although the APR is considered protective, chronic relative low-grade activation of the APR leads to a chronic inflammatory response that is known to play a role in the progression of OA (50). A key and common feature of all chronic pain conditions is long-term neuronal plasticity in pain-signaling circuits that result in increased neuronal responsiveness to their normal input and/or recruitment of a response to subthreshold input (51). Such changes are known to be driven by chronic inflammatory conditions, such as OA, which result in sustained, maladaptive inflammatory responses, in which pro-inflammatory mediators persistently activate and sensitize neurons at different levels of the nociceptive pathway (52).

Low grade inflammation is considered to play a role in the pathophysiology for CKD in cats. Work by Javard et al. (53) demonstrated that two APR proteins, SAA and haptoglobin, were significantly higher in cats with CKD suggesting that feline CKD is associated with systemic inflammation. Ferlizza et al. (54) completed a proteomic analysis of urine from cats with and without CKD and identified DAPs including RBP and proteins associated with coagulation, complement, and lipid metabolism. In cats with CKD, infiltration of inflammatory cells has been associated with tubulointerstitial fibrosis based on histological evaluation (55) supporting the finding of macrophage activation.

#### LXR/RXR activation & FXR/RXR activation

The LXR/RXR and FXR/RXR Activation pathways were found to be dysregulated in association with higher DJD and pain scores. These pathways are most commonly associated with the liver and intestine, but they are abundant and active in other tissues. As members of the nuclear receptor superfamily, LXR and RXR can form heterodimers with each other, and function as transcription factors that regulate many different physiological processes. Our findings are supported by studies that have reported the involvement or dysregulation of LXR/RXR pathways in OA tissue and synovial fluid (56–58). Further, in a recent study involving proteomic profiling of menisci from OA patients compared to healthy menisci, the LXR/RXR pathway was found to be significantly altered (59).

To date, no work has identified or evaluated the role of LXR/RXR or FXR/RXR pathways in CKD in cats. FXR activation has been reported to reduce inflammation and oxidative stress, both of which are closely associated with the pathophysiology of renal disease (60, 61). Whereas, LXR activation was shown to potentiate cellular inflammatory cytokine response, by activation of toll like receptor 2 and 4 and ERK 1/2 signaling pathways (62).

In addition to the inflammatory roles of these pathways, FXR/RXR and LXR/RXR may have further roles in CKD. FXR controls lipid metabolism by repression of sterol regulatory element-binding protein 1c (SREBP-1c) in liver (63), and in mouse models of streptozotocin induced diabetes and diet induced obesity, SREBP-1 was shown to be overabundant in the kidney where it promoted renal lipid accumulation and the expression of profibrotic factors which all contribute to kidney damage (64). FXR activation has been reported to reduce renal overexpression of SREBP-1 and lipogenic enzymes as well as decreasing lipid accumulation in mouse models of diabetes (65) and obesity (60), thereby acting to reduce profibrotic factors. Further, interstitial lipid deposits in the kidneys of cats with CKD have been reported (55) but the cause and impact on disease, while hypothesized, is not understood. FXR is highly abundant in the tubules of the kidney where it plays a key role in regulating water reabsorption by modulating urine volume, osmolarity, and regulating renal aquaporin 2. Chronic hypoxia is associated with oxidative stress and uremic toxins and has been shown to be an important contributing factor to the progression of CKD in cats (66). In the current cohort, we did not have data on the progression status of CKD in any of the cats—our data was cross-sectional at a single point in time. In a mouse ischemia reperfusion model, activation of FXR has been demonstrated to lower reactive oxygen species levels and protect kidney mitochondria by upregulating antioxidant pathways and controlling glutathione metabolism (61). Overall, our findings of dysregulated LXR/RXR or FXR/RXR in cats with CKD are consistent with the literature.

#### Complement system

C1Qb emerged as a key DAP common to the three major groupings of disease severity for DJD + Pain in our study. We showed that C1Qb, complement component 1r (C1r), complement component 4 binding protein alpha (C4BPA) and complement factor H (CFH) were upregulated, while complement component 8 subunit gamma (C8G) was downregulated. Our results indicated that both effector (C1Qb, C1r) and inhibitor [CFH/factor H and C4BPA/complement component 4 binding protein (C4bp)] proteins were increased, suggesting both augmented and modulated activity of the complement pathway, respectively. We hypothesize that inhibitor protein genes were upregulated to limit complement activation and maintain homeostasis. Complement activation has been implicated in inflammation and OA in studies of synovial fluid and tissues from OA joints and clearly supports a role of components of the complement system in OA (67–70). In one study (69) synovial fluid concentrations of complement component 3a (C3a) and complement component 5b (C5b) were increased, and C4BP and CFH decreased in human OA patients compared to controls. However, in our study, the inhibitory proteins (C4BP and CFH) were upregulated suggesting a compensatory response to limit overall activation of the complement pathway. Possible reasons for this disparity are that our samples were obtained from serum, which reflects activity from all joint tissues, and where homeostasis could play a greater role.

Certain components of the complement system have been linked to inflammatory pain, such as complement component 5a and C3a (71). Relatively little work has evaluated circulating complement factors in relation to OA and pain, and with respect to OA, most has been directed at identifying biomarkers of OA. However, serum concentrations of components of the complement system have been found to be significantly associated with OA in human patients (3, 72, 73). One such study did identify serum complement C3f as having significant correlation with parameters reflecting local inflammation and bone remodeling, as well as decrease in cartilage turnover (73). A recent serum proteomic study found complement component 3 (C3) was significantly elevated in human patients with radiographic knee OA, and further that serum C3 differentiated between OA and RA patients (72). However, other serum proteomic evaluations in human patients with knee OA did not identify the complement system as being significantly related to pain intensity (74), but did identify fibroblast growth factor-21, eukaryotic translation initiation factor 4E-binding protein 1, IL-6, macrophage colony-stimulating factor 1, and tumor necrosis factor superfamily member 12 as being related to pain intensity (74).

With respect to CKD, the majority of literature has focused on urinary proteomics. However, a recent study evaluated the proteome of serum samples from humans with terminal stage CKD and healthy controls found increased levels of complement system proteins (75). Our data showed a similar pattern of upregulation for C1Qb, C1r, CFH and C4BP and downregulation of C8G. While the complement pathway was not one of the top 5 pathways listed in Table 6, it was identified as being differentially abundant with a −log *p*-value of 1.40.

#### Coagulation system and intrinsic prothrombin activation system

The coagulation system and intrinsic prothrombin activation system were among the top canonical pathways dysregulated for DJD + Pain. Proteins upregulated in the coagulation system included factor XI (FXI), fibrinogen alpha chain (FGA), and von Willebrand Factor (VWF); downregulated proteins included factor XIIIb (FXIIIb). Several proteomic studies of synovial fluid, in both humans (56, 67, 76, 77) and horses (78) have shown dysregulation of the coagulation pathways in OA. However, changes in serum have not been reported. The upregulation of FXI in association with DJD + Pain supports dysregulation of the kallikrein pathway (79). When activated, subunit FXIa, along with FXIIa, cleave kininogen to release bradykinin (BK). BK has been implicated in the pathogenesis of OA in studies evaluating synovial fluid from human patients and mouse models (76). Our findings of downregulated FXIIIb in cats with DJD + Pain may be an indicator of greater activation of FXIII to FXIIIa, and enhancement of the fibrinogen pathway toward greater inflammation and enhanced activation of complement component 5 in the complement pathway. The finding of upregulation of VWF in our study is supported by studies looking at synovial fluid from OA patients (80). Similarly, upregulated FGA found in our study is supported by findings in human OA synovial fluid (70, 77).

#### LPS/IL-1 mediated inhibition of RXR function

The LPS/IL-1 Mediated Inhibition of RXR Function pathway was identified in connection with CKD in our study, but not DJD or pain. It plays a role in infection, inflammation, and injury and seems consistent with the inflammatory pathways and proteins we see in this analysis. Liu et al. (81) identified this pathway as being induced in mesangial cells in galactose-deficient IgA nephropathy based on mesangial cell proteomics and glomerular transcriptomics. Li et al. (82) and Xie et al. (83) both demonstrated the involvement of this pathway in acute kidney injury due to triptolide toxicity. Fatty acid binding protein 4 (FABP4) was upregulated in our analysis for this pathway. Expression of FABP4 has been demonstrated in the kidney of CKD patients and the expression is associated with renal dysfunction (84), while urinary FABP4 has been proposed as an early biomarker of kidney damage (85). In addition, high levels of circulating FABP4 have been demonstrated in patients with CKD (86).

#### IL-12 signaling and production in macrophages

Macrophages are known to play a key role in inflammation associated with CKD and the severity of infiltration in renal tissue from cats (focal infiltrate) is correlated with CKD progression and interstitial fibrosis (55, 87). RBP is synthesized by the liver and functions as a carrier protein for retinol (Vitamin A1). As a protein that is freely filtered and reabsorbed by proximal renal tubules, it has been suggested that it may provide an early indication of proximal tubular dysfunction. In dogs with X-linked hereditary nephropathy, urinary RBP4:urinary creatinine ratio progressively increased throughout disease progression and this correlated with serum creatinine, glomerular filtration rate, and irreversible histological damage (88). Segev et al. (89) demonstrated that urinary RBP4 increased in dogs with acute kidney injury as a result of heatstroke.

### Study limitations

One important limitation of this study is that a validation study was not performed. Often the “hits” identified on analysis of an initial cohort of patients/animals are not found on a second independent cohort. Future work should include performing a validation analysis on a separate cohort. Additionally, validation of differential expression of these proteins using other techniques would have been useful to add confidence to the findings. Bodyweight and BCS were not different between the groupings of pain/DJD severity, but excess fat may contribute to “inflammatory proteins”. BCS is a measure of excess body fat, but is extremely subjective and was assessed by different veterinarians in this study, and so considered inaccurate for use in analysis. Future studies should include objective or valid measures of body fat content to evalute the effect of “obesity” on proteomic data. Other uncontrolled biological factors may have influenced our data, such as genetic variability in the cats, and relatively small sample size in some severity categories. Slight variations in sample processing may have affected our results, although all samples were selected based on the fact they had not undergone any freeze thaw cycles. Our categorization of CKD as “positive” or “negative” was simplistic. This approach was taken for two reasons. Firstly, the data used to classify cats' CKD status was taken from a single point in time—cats had not been followed longitudinally to determine whether CKD was progressive. Also, blood pressure measures were not collected during the evaluation of these cats. Secondly, due to the constraints of the original studies regarding inclusion criteria, the majority of the cats with CKD (57 of the 67 cats) were IRIS stage 2; to have parsed out the classification into 3 groups, with low numbers, would have limited the statistical analysis options. The limited spectrum of severity of CKD and our inability to subcategorize the severity of CKD meant that we were not able to attempt to factor in the effect of renal dysfunction on clearance of small proteins. Further work should evaluate a larger cohort of cats with a broader spectrum of severity of CKD and define the progressive status of CKD in each cat. Despite the clear limitations on phenotyping cats for CKD, we felt it was important to define CKD status and use this in the analysis because of the known role of type II angiotensin II receptors in pain processes (90).

## Conclusion

The analysis of proteomics from a large number of well-phenotyped cats has provided a unique opportunity to study common dysregulated genes in degenerative joint disease (DJD), pain, and chronic kidney disease (CKD). This is the first study in any species to use serum samples from fully phenotyped subjects—every joint was imaged and every joint assessed for pain—and as such, provides a unique perspective on mechanisms. Using mass spectrometry-based proteomic profiling of the serum of 200 highly phenotyped cats with varying burdens of DJD, pain, and CKD, we identified significant individual proteins and pathways. Several central themes emerged, some of which have been previously underappreciated. Our data suggest dysregulation of liver proteins typically involved in lipid metabolism or transport and inflammation as playing prominent roles. In addition to LXR/RXR and FXR/RXR activation, functional pathway analysis based on DAPs across individual disease states (DJD, pain, CKD), identified APR signaling and the complement system as playing prominent roles. Similar pathways were identified with the added co-morbidity of CKD, along with the addition of IL-12 signaling and production in macrophages. Future work should evaluate differentially abundant proteins as potential biomarkers of disease (individually or as clusters). Further, these data can be leveraged to identify novel therapeutic targets to address the gap in our ability to manage DJD, pain, and CKD in cats. Finally, given that our work was in cats with naturally occurring DJD, and DJD is a significant health problem in humans, these results may have translational applicability to human health.

## Data Availability

The datasets presented in this study can be found in online repositories. The names of the repository/repositories and accession number(s) can be found in the article/Supplementary Material.
