# Supplementary figures and images for: Proteomic profiling of serum in cats with naturally occurring degenerative joint disease and co-morbid conditions
Source: Front Pain Res (Lausanne). 2025 Feb 4;6:1501932. doi: 10.3389/fpain.2025.1501932 (PMC11832531; doi:10.3389/fpain.2025.1501932)

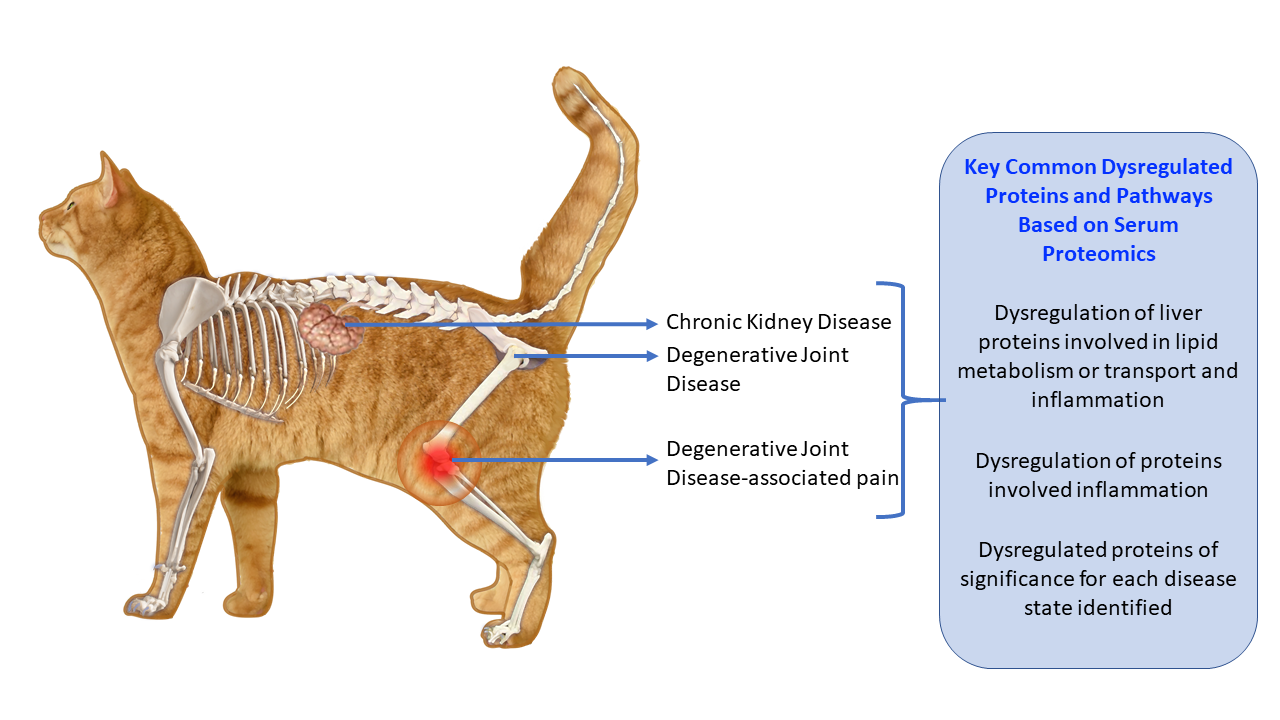

Supplement: Supplementary file 1 [file Image1.tif]
